# Evaluation of the standard procedure for the treatment of periprosthetic joint infections (PJI) in Germany - results of a survey within the EndoCert initiative

**DOI:** 10.1186/s12891-020-03670-y

**Published:** 2020-10-19

**Authors:** Christina Rimke, Andreas Enz, Hermann Josef Bail, Peter Heppt, Bernd Kladny, Gabriela von Lewinski, Christoph H. Lohmann, Katrin Osmanski-Zenk, Holger Haas, Wolfram Mittelmeier

**Affiliations:** 1 Orthopädische Klinik und Poliklinik, Universitätsmedizin Rostock, Doberaner Straße 142, 18059 Rostock, Germany; 2 Klinik für Orthopädie und Unfallchirurgie, Klinikum Nürnberg Süd, Universitätsklinik der Paracelsus Medizinischen Privatuniversität Nürnberg, Breslauer Straße 201, 90471 Nuremberg, Germany; 3 OCE Orthopädie Centrum Erlangen, Nägelsbachstraße 49A, 91052 Erlangen, Germany; 4 M&I Fachklinik Herzogenaurach, In der Reuth 1, 91074 Herzogenaurach, Germany; 5 Klinik für Unfallchirurgie, Orthopädie und Plastische Chirurgie, Universitätsmedizin Göttingen, Robert-Koch-Straße 40, 37075 Goettingen, Germany; 6 Orthopädische Universitätsklinik, Otto-von-Guericke Universität Magdeburg, Leipziger Str. 44, 39120 Magdeburg, Germany; 7 Allgemeine Orthopädie, Unfallchirurgie und Sportmedizin, Gemeinschaftskrankenhaus St Elisabeth St Petrus St Johannes gGmbH, Haus St. Petrus / Bonner Talweg 4-6, 53113 Bonn, Germany

**Keywords:** Revision arthroplasty, Two-stage revision, Periprosthetic joint infection, Certification, Incident reporting, Surveys

## Abstract

**Background:**

The periprosthetic joint infection (PJI) is a severe complication in the field of arthroplasty. Despite the rising number of primary joint replacements, no unified therapeutic standard has been established for the treatment of PJI yet.

**Methods:**

A survey on the principles of treatment of PJI in Germany was conducted. A total of 515 EndoProthetikZentren (EPZ) were included, resulting in a response rate of 100%.

**Results:**

For early infections 97.6% of the centers use prosthesis-preserving procedures (DAIR). A one-stage exchange was implemented by less than 50% of the centers. If implemented, this treatment entails a prior selection of patients for a successful treatment. The two-stage exchange is performed in all centers, and most centers proceed with the implantation of a cemented spacer between stages. 75% of the centers proceed with a center-based concept for the treatment of PJI.

**Conclusion:**

The aim of a uniform PJI standard at the centers has not yet been fully achieved. Further improvements within the certification were initiated. The most relevant treatment options in Germany are displayed. The two-stage revision with a cemented spacer is the most widely implemented treatment. This exposition of principles could help for the further development of standardized treatment guidelines and definitions.

## Background

Joint replacement in end-stage joint diseases has been a well established and globally widespread surgical treatment method. Approximately 448.000 joint replacements are carried out in Germany every year [1]. Periprothestic joint infection (PJI) may occur in 0.2–2% of primary joint arthroplasty and up to 9% in implantation of megaprotheses [2–4]. Due to demographic change and an increased functional demand of patients, an increase in primary implantation of endoprostheses is expected and thus an increase in the absolute number of PJI [5–7]. In implant loosening, infection already represents one of the most frequent indication for revision surgery. Incidence rates are up to 30%, in addition to loosening of aseptic prostheses [1, 8–10]. PJI are associated with challenging diagnostic and therapeutic procedures and is one of the most serious complications [7, 11, 12]. Embedded in biofilm, bacteria show a better survival rate and a significantly worse accessibility for antibiotics [13]. Therefore, explantation of the prosthesis is required in many cases for successful treatment of PJI. Possible treatment methods for PJI include prosthesis-preserving procedures (DAIR) as well as one- and two-stage revision [11]. The optimal treatment procedure for PJI is often discussed [11, 14]. To date, there is no internationally or nationally unified therapeutic standard for infected endoprostheses. The definition of such a therapy algorithm is crucial in order to guarantee successful treatment of PJI.

The EndoCert initiative was established in Germany in 2012 as the first worldwide certification system of medical centers for total joint replacement. The aim of this initiative is to maintain quality standards in primary and revision arthroplasties in large joints. The associated centers also develop and define standards as well as treatment processes, and they are subject to continuous re-certification [15, 16]. In the present study, the standards for the treatment of PJI in all EndoCert arthroplasty centers in Germany were assessed. The therapy algorithms for one- or two-stage exchange, the duration of the two-stage interval, and the concepts of implant anchorage were analyzed. Potential differences in the treatment concepts between centers were identified and analyzed with respect to their numbers of endoprosthesis revision operation. The results of this study should lead to a harmonisation of the procedure for PJI within the EndoCert certification process.

## Method

A questionnaire on the principles of treatment for PJI was developed in cooperation with the EndoCert Certification Commission. Questionnaires from all centers (*n* = 515) at the time of the survey (2015, recording years 2013–2014) were included in the evaluation (see supplementary file). The response rate of the survey was 100%. More than half of the arthroplasty centers in Germany are affiliated with the EndoCert initiative. The centers are subdivided into 73% endoprosthesis centers (EPZ) with at least 100 interventions per year and 27% endoprosthesis centers of maximum care (EPZmax) performing at least 200 endoprosthesis interventions including at least 50 replacements of implants per year.

The questionnaire consists of five questions regarding therapeutic options for septic endoprosthesis replacement. The multiple-choice questions could be supplemented with further individual information by the center. Multiple answers were possible for certain questions. The feedback represented a self-declaration of standards of the centers, their present implementation is annually checked by random surveys within the framework of the audits.

### Statistics

The data documentation and statistical evaluation of the collected data were carried out with Microsoft Excel 2013, version 15.0. The standard parameters of the descriptive statistics were determined and shown as absolute and percentage frequency. This is a complete survey since all of the centers which were certified at that time participated in the survey.

### Definition of periprosthetic joint infection

Certified endoprosthesis centers are subject to a published PJI definition (MSIS [17], ICM [18], Trampuz [19], HICARE [20]). The diagnostic procedure is carried out in accordance with the definition.

### Ethics approval

The study was approved by the local institutional ethical committee (A2015–0055).

## Results

A total of 515 certified centers were included. 97.6% of these perform a procedure like debridement, antibiotics, and implant retention (DAIR) in case of an early infection. Only 3 centers do not follow this concept and nine centers left this question unanswered. Most centers (30.5%) stated to perform DAIR up to 6 weeks after primary prosthesis implantation in case of early joint infections. 40.9% of the centers with more than 200 annual revisions choose this procedure up to a maximum of 4 weeks after implantation of the endoprosthesis, 50.4% of the centers use DAIR in an interval of 4–10 weeks. As part of the procedure 98.0% of the centers perform an exchange of the mobile parts of the implants.

If the DAIR procedure is not the appropriate treatment option, the one- and two-stage complete exchanges are carried out by the centers. All participating centers treating PJI use two-stage complete exchanges. However, only less than 50.0% of the centers regard the one-stage complete exchange as a possibility to treat PJI. The use of the one-stage exchange is predominantly implemented in individual cases (Table 1). The most relevant criteria for the centers to perform this procedure is the early infection (75%) (Table 1). Regarding these results, it has to be considered that in 9.7% of the questionaries question number two was not correctly answered, the question in the related questionnaires was therefore not included in the evaluation.

**Table 1:** Performance of the one-stage exchange in the hip and knee; conditions for the implementation of the procedure

	hip (%)			knee (%)		
	n	carried out	not carried out	n	carried out	not carried out
EPZ total	495	48,7	51,3	485	44,0	56,0
EPZ	357	45,1	54,9	349	39,5	60,5
EPZmax	138	58, 0	42,0	136	55,1	44,9
**Implementation**						
early infection	76,8			73,7		
tissue damage	31,1			30,0		
patient’s age	31,1			29,6		
germ spectrum	29,0			30,0		
others	24,1			26,8		

**Fig. 1:**
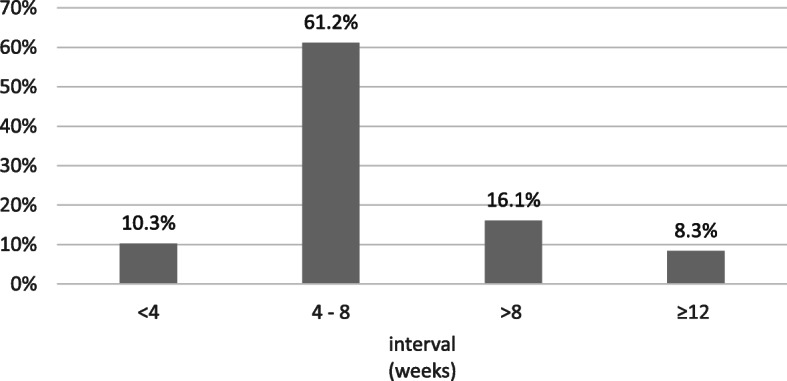
Interval in the two-stage exchange (grouped) (*n* = 515)

All centers perform a two-stage complete exchange, but with different duration of the interval. The intermediate interval data ranged from 4 to 120 days and was divided into 4 groups (Fig. 1). The largest proportion of the centers choose an interval duration between 4 and 8 weeks. In addition, 90.0% of the centers use cement spacers during the intermediate interval. During the explantation procedure most centers (58.4%) take 5 or more tissue samples for further diagnostics. 2 to 4 samples are taken by 37.9% of the centers, 3.7% take 1 sample or did not specify their answer. Only half of the centers (52.3%) take these samples from standardized localizations.

**Fig. 2:**
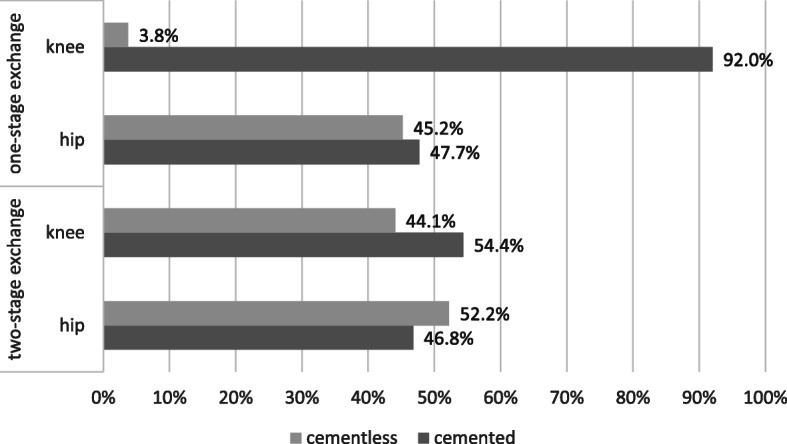
Choice of implant fixation in one- and two-stage exchange for re-implantation (one-stage exchange: hip: *n* = 241; knee: *n* = 213; two-stage exchange: hip: *n* = 515; knee: *n* = 515)

A further distinction between the one- and two-stage revisions was the final implant fixation (Fig. 2.). The centers that perform single-stage replacement in the knee almost entirely opt for cemented implant fixation in the knee (cementless 3.8%, cemented 92.0%). In contrast, for two-stage revision, these centers decide 10 times more in cementless implant fixation in the knee (cementless 44.1%, cemented 54.4%). For revisions procedures at the hip joint, the centers perform a cementless procedure (52.2%) more often than a cemented procedure (46.8%). When using a cemented fixation, 70.7% of the centers choose a prefabricated cement compared to 19.6% using an individually mixed type only.

Seventy-five point zero percent (*n* = 384) of the hospitals follow a specific concept to treat PJI (Table 2). This question was not answered by 16 centers (3.0%).

**Table 2:** Presence of a defined clinical concept and type of diagnostic before re-implantation differentiated by the annual number of exchanges performed in the center

center-internal concept	EPZ (total) (*n* = 515;%)	< 50 exchanges/year (*n* = 293;%)	50–100 exchanges/year (*n* = 138;%)	101–200 exchanges/year (*n* = 61;%)	> 200 exchanges/year (*n* = 23;%)
existing	75,0	72,3	82,0	83,6	87,0
non-existing	22,0	27,7	18,0	16,4	13,0
**Type of diagnostic before reimplantation**					
none	12,4	10,2	15,2	16,4	13,0
joint puncture	61,0	60,1	61,6	65,6	56,5
microbiology	23,1	29,4	13,8	18,0	13,0
microbiology & histology	36,7	36,5	37,0	36,1	39,1
no answer	0,6	0,7	0,7	0,0	0,0

Centers with a low number of annual revision surgeries apparently use preset algorithms less frequently. Hospitals with more than 200 exchange operations per year have reproducible standards for the treatment of PJI – twice as much than those with less than 50 exchange operations per year.

The results for the type of diagnostic are shown in Table 2. Especially large centers make use of microbiological and histological diagnostics before re-implantation more frequently than the total of all centers.

**Fig. 3:**
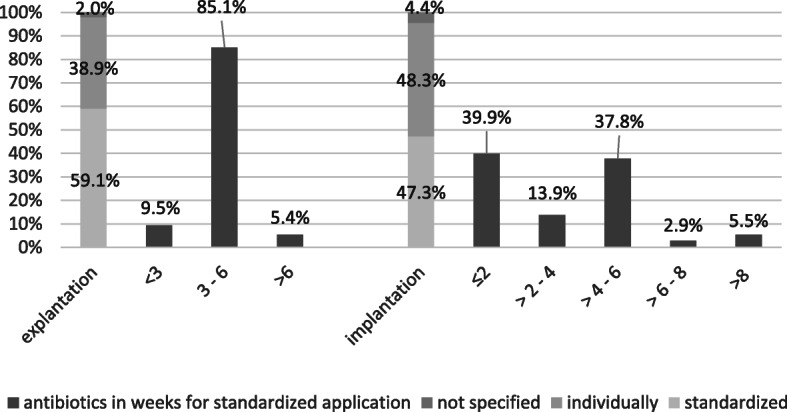
Way of application of antibiotics after explantation and re-implantation; Duration of application of antibiotics in weeks for standardized application

A standard of additional antibiotic therapy is used only in 59.1% of the centers after explantation and only 47.3% after insertion of new implants (Fig. 3). After explantation 86.0% hospitals apply antibiotics over 4–6 weeks, while after reimplantation the duration is more inhomogeneous.

## Discussion

The aim of this study was to present the current status of algorithms for the treatment of PJI in certified arthroplasty centers. All german centers certified by EndoCert have been included. These 515 centers are representing the most of the high-volume arthroplasty centers regarding the THR’s and TKR’s including the respective revisions [1, 15]. All EndoCert approved centers are obliged to send their implant data of primary and revision procedures to the German joint register EPRD. When comparing the register data with the knowledge of center-specific algorithms, these results may offer successful approaches for the treatment of PJI in the future. This is one of the few and one of the most comprehensive national studies on treatment of PJI [21–23].

The response rate of the survey questionnaires was 100%. Only question two was not fully evaluable with 90.3% and did not reach the maximum overall level; all other questions were answered with a 100% evaluable response rate.

In the registers, such as the Swedish Hip and Swedish Knee Arthroplasty Register (SHAR and SKAR [24, 25]), the American Joint Replacement Registry (AJRR [26]), the Australian Orthopedic Association National Joint Replacement Registry (AOANJRR [27]), as well as in the German joint register (EPRD [28]), the term revision is not consistently defined. There is no definition if a revision is reported as implant-related or if it is considered infected. Infections are recorded as the cause of revisions but are not further discussed in detail. The change of inlays in the case of infections does not count as revision in the registers and is not further evaluated with respect to success or failure of the procedure; soft tissue surgery is not recorded in some cases either [25]. Only the SKAR addresses this problem in a separate section [25]. Revision procedures and algorithms are not recorded in any of the registers [24–26, 28].

Due to the lack of uniform therapy algorithms for the treatment of PJI, the selected procedure is often based on traditions, experience and preferences of the surgeon and the institution [5, 29]. While standards in primary joint replacement must already be established at the time of the initial certification of the center, the goal of consistent, reproducible and clinically coordinated diagnostic and therapeutic processes in treatment of PJI has not yet been sufficiently achieved. There is a development towards standardization in the field of septic endoprosthetic cases, at least in the certified centers. The high number of centers (*n* = 115) without a defined therapy concept clearly illustrates the need of compulsory and approved standards. Within the complication management the EndoCert centers had to define an intraclinical treatment concept which is reviewed within the scope of regular audits. On an international and national level, this issue is of great importance [30] and a standardized approach is necessary when treating PJI.

This study showed that almost all EndoCert centers (97.6%) use DAIR in early PJI. In addition to joint debridement, the replacement of modular prosthetic components is an essential part of the concept (98%) in order to eradicate a potential source of infection and enable more extensive debridement [31]. A large number of centers stated that they would perform this procedure within 6 weeks after initial implantation while retaining the implant components that are well fixed in the bone. The literature states that after 6 weeks, the chance of success of implant-preserving surgery is reduced to 40% [32]. This is in agreement with the findings of implant-associated biofilm research showing that biofilm forms on the biomaterial surfaces early after implantation and is particularly advanced after 4 weeks [33, 34]. In centers with more than 200 revisions per year, partial replacement is used up to a maximum of 4 weeks, or in an interval of 4–10 weeks after primary implantation. The use of DAIR in cases of late hematogenous endoprosthesis infections, which can impress like an early infection with a fulminant course, was not considered in this survey, but represents a further treatment strategy.

In case that DAIR is not effective, all centers change their strategy to one- or two-stage revisions of all implant parts. The significance of both procedures in PJI is intensely discussed; some studies describe similar or higher success rates for one-stage revision [14, 35, 36]. Patient selection for eligibility of the procedure precedes the one-stage exchange. This is crucial for the success of the therapy [37–39]. The present study has shown that patient selection in the case of a one-stage revision is also of great significance for the EndoCert centers. The definition of early and late infection is not uniformly applied [11, 31]. There is no clear statement on a duration regarded as early infection by the centers and the corresponding period to perform the one-stage exchange. In order to be able to determine uniform therapy algorithms, it is crucial to define early infection. The one-stage exchange is only conducted at half of the EPZ for single cases and is not regarded as a 100% equivalent alternative to the two-stage procedure.

The two-stage exchange is implemented in 100% of the EPZ in Germany. This procedure can be applied to a wider range of patients and leads more reliably to successful treatment of the infection, especially in cases with difficult-to-treat bacteria [20, 40]. Shorter OR time, the possibility of fractional expansion, management of the infection situation, the systematic administration of appropriate antibiotics both intravenously and locally with antibiotic-reinforced spacers, and an interval to reduce the risk of infection persistence prior to reinstallation result in an increased use in the EPZ. In cases of severe infections, poor soft tissue conditions or (partially) resistant bacteria, the literature reports a clear advantage of a two-stage procedure [41]. A disadvantage is the repeated anesthesia and relatively limited mobility in the (spacer) interval. The duration of the implant-free interval is 4 to 8 weeks in 61% of the centers and corresponds to the reports in the literature [20, 29, 42, 43]. The advantage of the two-stage revision is the shorter duration of the individual procedures and the possibility of obtaining several delayed tissue samples from different areas of the wound region, resulting in early and better detection of infection and appropriate administration of antibiotics [20].

The duration of the implant-free interval showed a high discrepancy of the indicated durations of 4 days to 120 days; a further differentiation of the values could not be determined from the questionnaire. On average, the spacer was in place for 42 days before the next revision, corresponding to the time recommended in the literature [20, 42].

PMMA spacers containing antibiotics are used during the interim interval in more than 90% of the centers and the Girdlestone situation is also implemented in 23% of the centers as alternative solution. If PMMA is used, the hospitals applied individually shaped cement spacers (61%) and pre-formed spacers (42% of the centers). When inserted in the knee and hip, this spacer should increase mobility in the interim phase [44]. However, the cast spacer can only be used for minor bone defects, while individual spacers are adapted to the specific defects and can bridge larger distances and ensure the release of antibiotics in the overall defect [45]. It is discussed whether the movement of the spacer has favorable properties for the healing of the infection [46, 47].

In addition to surgical decontamination, the management of suitable antibiotics plays an important role in the treatment of infections. The duration of therapy as stated in the literature varies from 14 days to 3 months [20, 29, 42, 48]. A distinction between removal and replacement is advisable and is carried out by 94% of the centers. Two hundred eighty hospitals (45.6%) choose an individual application of antibiotics. At the time of the survey, the rate of standardization was still very low, which could partly be due to the various literature. The duration of antibiotic treatment after removal of the prosthesis is largely homogeneous; 85% of the centers surveyed state a duration of 3 to 6 weeks and only 5% state a longer duration of treatment. In contrast, the centers are much more inconsistent after reimplantation. The majority (39.9%) stated a duration of antibiotics of up to 2 weeks whereas 37.8% stated a duration of 4 to 6 weeks. Further prospective comparing studies are necessary to determine the best outcome of the treatment concepts. As the duration of the course of antibiotics increases, the antibiotic-associated rate of adverse side effects that pose a risk to the patient increases [49].

## Conclusion

The rising number of primary joint replacements underlines the importance of developing standards and harmonized definitions for the treatment of PJI. These standards have not yet been fully achieved neither in Germany nor internationally. The most relevant treatment options in Germany are shown. The two-stage revision with cemented spacer is the preferred method in PJI treatment, one-stage revision is performed in the centers under strict indication and for selected patients. This summary of principles could serve as a basis for the further development of standardized treatment guidelines and definitions.

## Supplementary information

**Additional file 1.** “Principles of treatment for septic endoprosthesis revisions”, endoCert Questionnaire for Principles of treatment for septic endoprosthesis revisions for the endoprosthetic centers.

## Data Availability

The consent of EndoCert – an Institution of the german socitey of orthopaedics and orthopaedic surgery has been granted. The data was collected and evaluated within the scope of the EndoCert certification. The specially designed questionnaire and the data obtained are stored and available at EndoCert.

## Abbreviations

AOANJRR: Australian Orthopedic Association National Joint Replacement Registry
AJRR: American Joint Replacement Registry
DAIR: Debridement, antibiotics, irrigation, and retention of the prosthesis
EPRD: Endoprothesenregister Deutschland (German joint register)
EPZ: Endoprosthesis centers
EPZmax: Endoprosthesis centers of maximum care
OR: Operating room
PJI: Periprothestic joint infection
PMMA: Polymethylmethacrylat
SHAR: Swedish Hip Arthroplasty Register
SKAR: Swedish Knee Arthroplasty Register
