# Supplementary material for: Evaluation of the standard procedure for the treatment of periprosthetic joint infections (PJI) in Germany - results of a survey within the EndoCert initiative
Source: BMC Musculoskelet Disord. 2020 Oct 19;21:694. doi: 10.1186/s12891-020-03670-y (PMC7574524; doi:10.1186/s12891-020-03670-y)
Supplement: Supplementary file 1 — Additional file 1. “Principles of treatment for septic endoprosthesis revisions”, endoCert Questionnaire for Principles of treatment for septic endoprosthesis revisions for the endoprosthetic centers. [file 12891_2020_3670_MOESM1_ESM.pdf]

Principles of treatment for septic endoprosthesis revisions

Type of endoprosthetics center	Registration number
☐ EPZ ☐ EPZmax	**EPZ - -**

1. Procedures to retain the prosthesis

The endoprosthesis is maintained postoperatively until ____ weeks after implantation at the latest.

☐ without changing parts ☐ with changing parts (e.g. PE-Inlay)

Comment:

2. Single-stage septic revision Multiple answers possible

2.1 Procedure with single-stage revision

☐ Single-stage revisions are not carried out

☐ Single-stage revisions take place at:

- ☐ early Infection
- ☐ depending on defect
- ☐ age-dependent
- ☐ others:

2.2 Implants for single-stage revision

☐ mainly cementless
☐ mainly cemented (with ready-made cement mixture)
☐ mainly cemented with individual Admixture of antibiotics to cement
☐ others:

3. Two-stage septic revision Multiple answers possible

3.1 Procedure for a two-stage revision

Interval: ___ Days (time between prosthesis removal and reimplantation)

Spacer after removal
☐ no spacer (Girdlestone)
☐ Metal spacer
- ☐ one-part
- ☐ multi-part

☐ Cement spacer
- ☐ individually formed
- ☐ Spacer over mould

Implant selection for the final restoration for a two-stage revision (reimplantation)
☐ mainly cementless
☐ mainly cemented
☐ mainly hybrid

3. Two-stage septic revision Multiple answers possible

Implant choice: If cement is used

- ☐ Cemented (with ready-mixed cement)
- ☐ mainly cemented with individual Admixture of antibiotics to cement

Special concept of the clinic

- ☐ is available
- ☐ is not available

Insofar as bone augmentation is performed

- ☐ Allograft
- ☐ Ceramic bone graft substitute material
- ☐ Combination of both concepts
- ☐ Admixture of antibiotics

comment:

4. Removal of the endoprosthesis

4.1 Procedure for taking samples

☐ number of samples: ___

☐ scheme of the sampling sites: ☐ yes ☐ no (see appendix: Example scheme HiCare)

4.2 Duration of the use of postoperative antibiosis

☐ after removal: ___ days ☐ after implantation of the new endoprosthesis: __ days

☐ always individual after removal ☐ after implantation of the new endoprosthesis always individually

comment:

5. Sample before reimplantation?

Do you take biological material for infection diagnosis after removal and before reimplantation?

- ☐ no
- ☐ yes: punction
- ☐ yes: microbiology - sample (tissue sample)
- ☐ yes: microbiology - + histology - sample

6. Please name the following persons: Multiple answers possible

Manager EPZ:

Coordinator EPZ:
